# A Case of Multiple Posterior Intercostal Artery Common Trunks in Conjunction with Additional Arterial Variations

**DOI:** 10.1155/2021/7430752

**Published:** 2021-11-18

**Authors:** Nicholas R. Fanselow, Nolan Wallace, Daniel Sehi, Lokesh Coomar, John Martin, Yun Tan, Daniel T. Daly

**Affiliations:** Center for Anatomical Science and Education, Department of Surgery, Saint Louis University School of Medicine, Saint Louis, MO 63104, USA

## Abstract

Several thoracic vasculature variations were observed in an 81-year-old male cadaver during routine dissection. These included 5 common trunks of posterior intercostal arteries, a descending branch of the right vertebral artery, and atypical neurovascular relationships within intercostal spaces. On the right side, two common trunks of posterior intercostal arteries were observed supplying the 4th-7th intercostal spaces and 9th-11th intercostal spaces, respectively. There was also a small accessary branch supplying the 9th intercostal space. The first three posterior intercostal spaces on the right were supplied by a descending branch of the vertebral artery. On the left side, three common trunks of posterior intercostal arteries were encountered, supplying intercostal spaces 3-5, 6-7, and 11 plus the subcostal space. An atypical neurovascular relationship was observed in the right 6th intercostal space, as well as the left 2nd, 3rd, and 6th intercostal spaces. This is the first case report that presents 5 common trunks of posterior intercostal arteries, as well as common trunks in conjunction with other arterial variation in the posterior thoracic wall. These variations carry a high level of clinical significance and may be helpful in guiding decision-making related to surgical procedures related to the posterior thoracic cavity and spine.

## 1. Introduction

The principal arterial supply of the posterior thoracic wall typically comes from 11 pairs of posterior intercostal arteries (PIAs) and one pair of subcostal arteries. The first two PIAs arise from the highest intercostal artery, a branch of the costocervical trunk [1]. PIAs 3-11 and the subcostal artery arise directly from the thoracic aorta (TA), in a segmental pattern [2].

PIAs typically travel in the superior aspect of the corresponding intercostal space (ICS) near the inferior border of their respective rib. The origin of these arteries is slightly caudal to where the PIAs are situated in the ICS thus requiring the PIA to travel superiorly and laterally along the lateral aspect of the vertebral column before reaching the costal groove on the inferior border of the corresponding rib [3]. Occasionally, PIAs run dorsally between the neck of the rib and vertebral transverse process, through the costotransverse foramen [4]. The typical organization of the intercostal neurovascular structures within the ICS is intercostal vein, artery, and then nerve from superior to inferior [5–7].

As the PIA travels in the ICS, it divides into two major divisions, which in turn gives off minor branches to supply numerous structures. These branches supply structures such as intercostal, pectoral, serratus, and deep back muscles, as well as the spinal cord and associated nerve roots, mammary glands, and skin [8, 9]. The right bronchial artery, which supplies areas of the right lung, often originates from the 3rd PIA on the right side [10–12].

**Figure 1:**
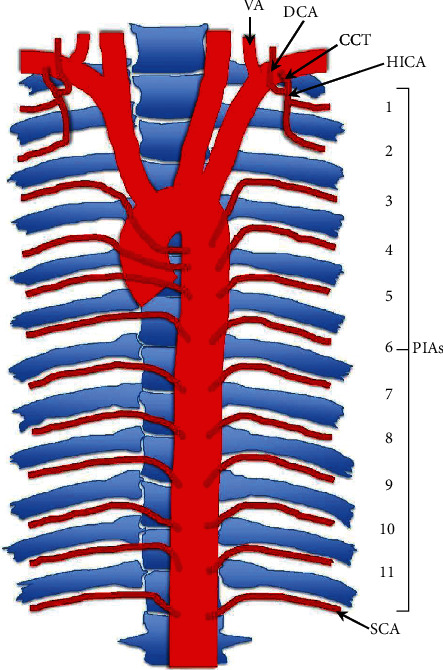
Graphic representation of typical arterial branching pattern of the posterior thoracic wall. VA: vertebral artery; DCA: deep cervical artery; CCT: costocervical trunk; HICA: highest intercostal artery; PIAs: posterior intercostal arteries; SCA: subcostal artery.

Although the pattern described above is that which is most observed (Figure 1), there is a great deal of variation in the arterial branching pattern within the area. The most frequent variations of the PIAs include those involving distance or spacing between PIA pairs, the absence of a PIA, division of a single PIA, or common trunks (CTs) of PIAs [13].

The prevalence of two or more CTs has been reported to be as high as 70% [8]. Higher numbers of CTs are less likely to be seen, and more than 4 CTs is a rare phenomenon. CTs are more frequent in the upper ICS (20-50%) than in the lower ICS (10-15%) [8].

The current report describes a case of bilateral variation in the PIAs of an individual with 5 CTs. This case involved other arterial anomalies, including variation in the supply of the most superior ICS, as well as variation in the neurovascular organization within the ICS. This case is of high interest for its rarity and clinical implications.

## 2. Case Presentation

An 81-year-old male body was received through the Saint Louis University Gift of Body Program of the Center for Anatomical Science and Education (CASE) with signed informed consent from the donor. The CASE gift body program abides by all rules set forth by the Uniform Anatomical Gift Act (UAGA).

**Figure 2:**
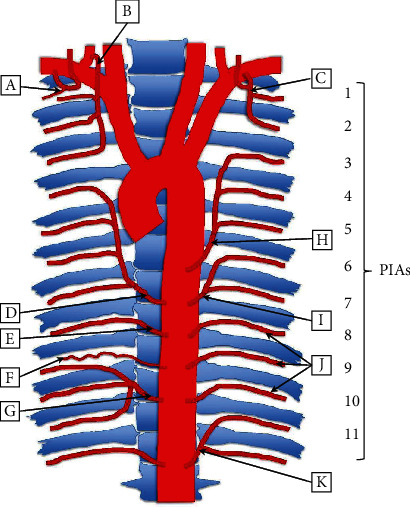
Graphic representation of variations of arterial branching pattern of the posterior thoracic wall, as seen in an 81-year-old cadaver: (a) right highest intercostal artery, supplying only 1st ICS; (b) right descending branch of vertebral artery, supplying 1st-3rd ICS; (c) left highest intercostal artery, following typical anatomical arterial supply; (d) right superior common trunk, giving off 4th-7th PIAs; (e) right 8th PIA, directly from the TA; (f) right 9th PIA, with diminished size directly from TA; (g) right inferior common trunk, giving of 9th-11th PIAs; (h) left superior common trunk, giving off 3rd-5th PIAs; (i) left middle common trunk, giving off 6th-7th PIAs; (j) left 8th, 9th, and 10th PIAs, directly from the TA; (k) left inferior common trunk, giving off 11th PIA and subcostal artery.

Multiple arterial variations in the posterior thoracic region were noted during routine dissection (Figure 2). The TA gave rise to 5 asymmetrical CTs of PIAs. A descending branch of the vertebral artery, as well as variation in the neurovascular organization within several ICS, was also noted.

### 2.1. Common Trunks

**Figure 3:**
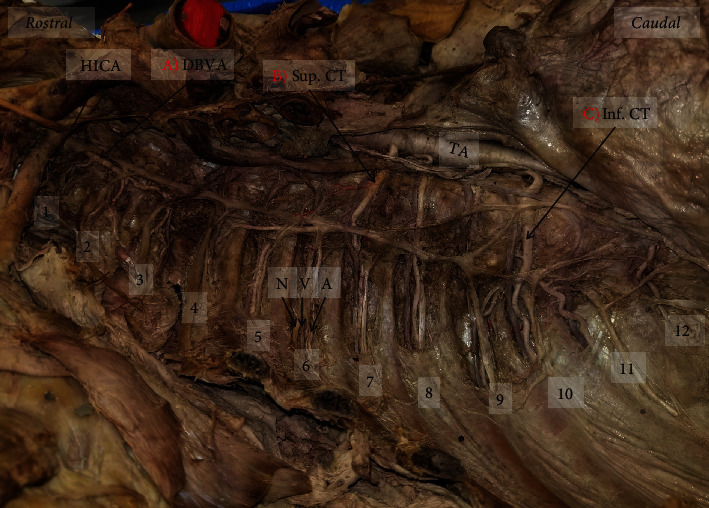
Right posterior thoracic wall with exposed intercostal spaces: (a) the descending branch of the vertebral artery supplying the 1st-3rd ICS; (b) superior common trunk arising from TA between7th and 8th thoracic vertebrae supplying 4th-7th ICS; (c) inferior common trunk arising from TA between 10th and 11th thoracic vertebrae supplying 9th-11th ICS. Numbers 1-11: ICS number; 12: subcostal space; HICA: highest intercostal artery; DBVA: descending branch of the vertebral artery; Sup. CT: superior common trunk; TA: thoracic aorta; Inf. CT: inferior common trunk; N: nerve; V: vein; A: artery.

On the right side of the thoracic wall, 2 CTs were observed arising from the TA, along with 2 typical PIAs. The right superior CT arose from the TA between the 7th and 8th thoracic vertebrae (Figures 2(d) and 3(b)). Near this location, the CT gave off a branch to supply the 7th ICS before continuing superiorly and laterally along the vertebral column. Along its path, the CT gave off 3 more PIAs which supplied the 6th, 5th, and 4th ICS before the CT terminated between the 4th and 5th thoracic vertebrae.

The right inferior CT originated from the TA at the level between the 10th and 11th thoracic vertebrae (Figures 2(g) and 3(c)). This CT traveled a short distance laterally before it split into 9th, 10th, and 11th PIAs supplying their respective ICS. These 3 PIAs also appeared to take on a more tortuous appearance than is typically seen. The 8th and 9th PIAs originated from the TA in the typical fashion and traveled to the respective ICS (Figures 2(e) and 2(f)). However, the 9th PIA was greatly reduced in size compared to the rest of the PIAs in the area, likely resulting from the dual supply of the 9th ICS.

**Figure 4:**
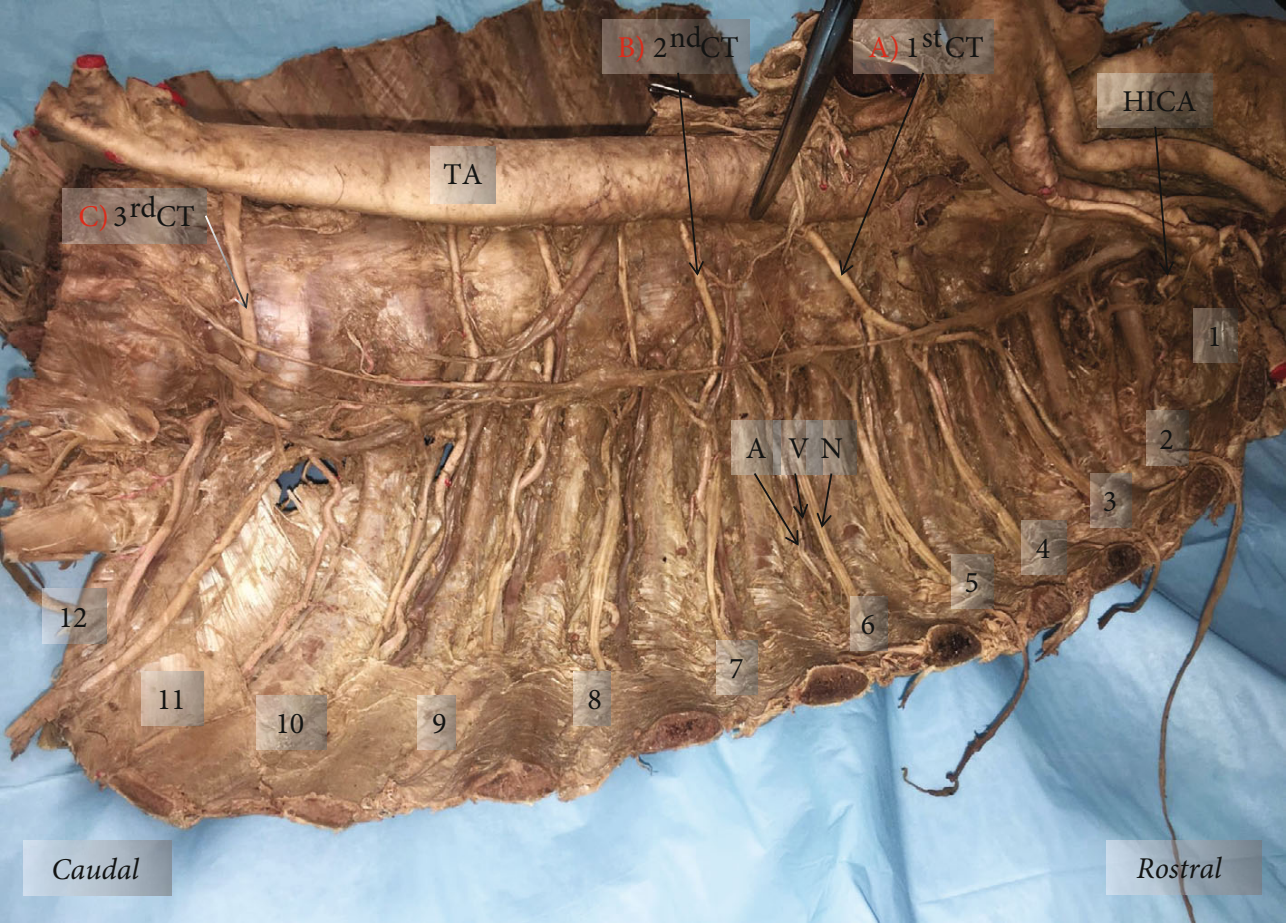
Left posterior thoracic wall with exposed ICS: (a) superior common trunk arising from TA between 6th and 7th thoracic vertebrae supplying 3rd-5th ICS; (b) middle common trunk arising from TA between 7th and 8th thoracic vertebral level supplying 6th-7th ICS; (c) inferior common trunk arising from TA between 12th thoracic and 1st lumbar vertebrae supplying 11th ICS and subcostal space. Numbers 1-11: ICS number; 12: subcostal space; HICA: highest intercostal artery; 1st CT: first (superior) common trunk; 2nd CT: second (middle) common trunk; 3rd CT: third (inferior) common trunk; TA: thoracic aorta.

On the left side, 3 CTs were observed arising from the TA, along with 3 typical PIAs. The left superior CT originated from the TA between the 6th and 7th thoracic vertebrae where it then coursed superiorly to supply the 5th, 4th, and 3rd ICS (Figures 2(h) and 4(a)). The middle CT originated from the TA between the 7th and 8th thoracic vertebrae before supplying the 7th and 6th ICS superiorly (Figures 2(i) and 4(b)). The left inferior CT arose from the TA between the 12th thoracic vertebra and the 1st lumbar vertebra before splitting into the 11th PIA and the subcostal artery (Figures 2(k) and 4(c)).

**Figure 5:**
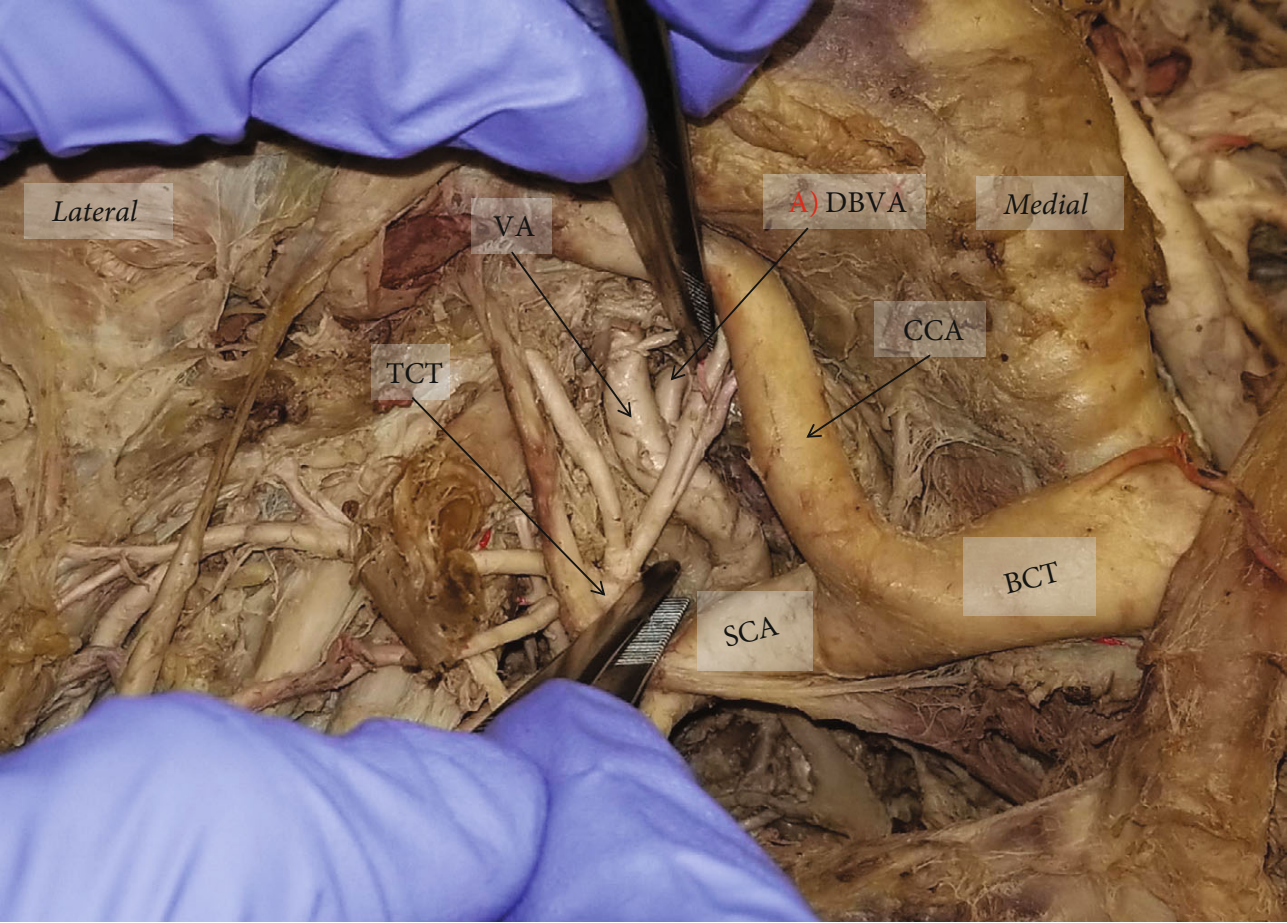
Right subclavian artery and presence of descending branch of the vertebral artery: (a) descending branch of the vertebral artery from its origin. TCT: thyrocervical trunk; VA: vertebral artery; DBVA: descending branch of the vertebral artery; SCA: subclavian artery; CCA: common carotid artery; BCT: brachiocephalic trunk.

The 8th, 9th, and 10th ICS were supplied by typical PIAs arising directly from the TA (Figures 2(j) and 5). Although these PIAs originated and traveled to the corresponding ICS, the 9th and 10th took on a more tortuous course than is typically seen. The 1st and 2nd ICS were supplied by the highest intercostal artery, as is normally described above (Figures 2(c) and 4).

### 2.2. Descending Branch of Vertebral Artery

**Figure 6:**
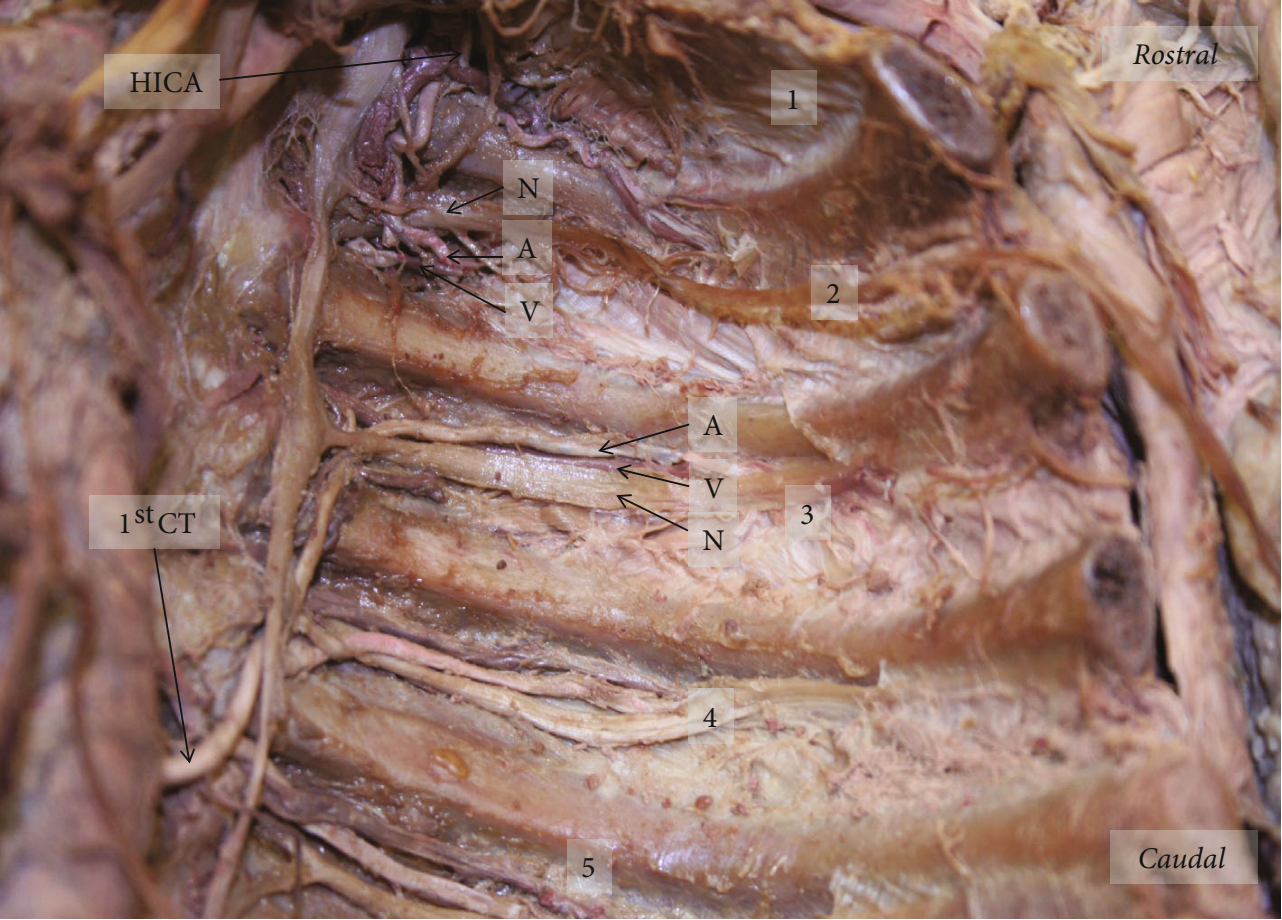
Left superior posterior thoracic wall showing neurovascular relationship in 2nd and 3rd ICS. The order of neurovascular bundle at the 2nd ICS (nerve, artery, vein) and 3rd ICS (artery, vein, nerve) from superior to inferior. Numbers 1-5: ICS number; HICA: highest intercostal artery; 1st CT: first common trunk; N: nerve; V: vein; A: artery.

Additional variations were noted on the right side of the thoracic cavity. A descending branch of the vertebral artery (DBVA) was found traveling inferiorly and dorsally near the first three ribs, through the costotransverse foramina (Figures 2(b), 3(a), and 5(a)). This DBVA gave off branches to supply the 1st, 2nd, and 3rd ICS on the right side. The highest intercostal artery was still present on the right side; however, it only supplied the 1st ICS, providing dual supply to this space (Figures 2(a) and 3). The left side of 1st and 2nd ICS was supplied by the left highest intercostal artery as normally seen (Figures 2(c) and 6).

### 2.3. Atypical Intercostal Neurovascular Bundle

There was an irregular relationship in the neurovascular bundle of the 6th ICS on the right side (Figure 3). The order observed was intercostal nerve, vein, and artery from superior to inferior. Additional variations were seen in the neurovascular bundle relationships of the left side in the 2nd (nerve, artery, vein), 3rd (artery, vein, nerve), and 6th (nerve, vein, artery) ICS (Figures 4 and 6).

## 3. Discussion

Only a few comprehensive reviews and case reports are presented in the literature on the topic of CTs of PIAs [4, 8, 13–15]. While cases have been reported, the current case seems to stand out among the rest for its rarity and clinical significance.

First, this case carries high significance due to the extreme rarity with the high number of posterior intercostal CTs, the presence of PIAs arising from the vertebral artery, and an atypical order of intercostal neurovascular bundle. This case provides an interesting backdrop to study the development and supply of the arterial structures within the posterior thoracic wall.

Development of the intercostal arteries begins early during the embryologic process and follows the formation of the endocardial heart tube. The endocardial heart tube forms during the 3rd and 4th week of development and is derived from splanchnic mesodermal cells in the region of the primary heart field [16, 17]. This endocardial heart tube forms an atrial outflow tract that consists of an aortic sac and 6 pairs of aortic arch arteries. Some of these arches connect the aortic sac with the right and left dorsal aortae, which are also derived from the aortic sac [17]. The right and left dorsal aortae travel inferiorly, dorsal to the primitive gut. Their distal ends then fuse between the levels of the fourth thoracic and fourth lumbar somite segment, forming the descending aorta [17, 18]. At the end of the third week of development, around 30 paired branches develop off the dorsal aorta to form the intersegmental arteries that carry blood to the developing somites and their derivatives [16, 17, 19]. Within the fully developed human, the intersegmental arteries within the thorax persist as the intercostal arteries [16, 19, 20]. Several intersegmental arteries on either side fuse in the neck to form the vertebral arteries [16, 20]. During development, improper fusion of intersegmental arteries within the thorax is likely the mechanism causing the formation of CTS of intercostal arteries, such as in the case presented above.

Second, based on the observed literature, this is the first case report of 5 CTs present in one individual. Cases with up to 4 CTs have been found and presented, but even these seem to be rare phenomena [8]. It has been reported that CTs are more frequent in the upper ICS than in the lower ICS, and the most frequent site of origin occurs at the level of the 3rd ICS or between the 3rd and 4th ICS [8, 21, 22].

The CTs presented in this case supply up to 4 ICS, giving a wide distribution of blood supply from a single CT. Once again, this may also be seen as a rarity, as most CTs supply fewer number of ICS [8]. Outside of these variations within the branching pattern of the PIAs themselves, this individual also had a few other anatomical anomalies.

The DBVA supplied the first three ICS on the right side in a pattern similar to reports presented in the literature; however, those reports were not associated with any posterior intercostal CTs [23, 24]. In this case, there were also 4 instances of the neurovascular bundle relationship within the ICS being abnormal. All these variations, within an individual, make the case of high relevance to study the posterior thoracic wall due to its unique nature. Understanding these relationships are of great importance in the setting of surgical procedures, such as thoracocentesis [25, 26].

Third, the increased number of variations in the current case demonstrates the variability of the blood supply to the posterior thorax. Under normal circumstances, a single pair of PIAs supplies one ICS and the surrounding structures that developed segmentally, such as the spinal cord and spinal roots. In these conditions, the chance of ischemic injury to the distributions of PIAs is relatively low due to collateral circulation between PIAs [27]. When a CT is present giving rise to multiple PIAs, this single CT now has a very wide range of distribution for its blood supply. If this CT was to become compromised, the chance of ischemic injury would be greatly increased, causing widespread consequences along the CT's path [8, 9]. Outside of this, the blood supply to essential organs, such as the lungs, may be disturbed as well. Since the right bronchial artery, which supplies the right lung, typically branches off the right 3rd PIA, any variation within the right 3rd PIA may carry consequences for the blood supply to that region [10–12]. For instance, in the current case, the 3rd PIA branched from the right DBVA which may have had downstream effects on the right bronchial artery.

Finally, the clinical implications of knowing the variations of CTs of PIAs are critical for interventional radiologist and clinicians performing procedures within the posterior thoracic wall, such as intercostal nerve blocks, thoracentesis, thoracotomy, thoracic aortic aneurysm repair, and posterior trunk reconstruction [28–34]. Since this is an area that can potentially have great variation in blood supply, it is essential to understand branching patterns before such procedures are performed. The more proximal areas of PIAs are also not protected by the ribs as much as distal regions, and this may be exacerbated in the case of CTs [35–37]. For these reasons, multiple sources have recommended using ultrasound to visualize PIAs before procedures are performed within the posterior thorax [33–35, 38, 39].

## 4. Conclusion

The case of multiple PIAs arising from CTs in conjunction with a DBVA and disruption of the neurovascular relationship within 4 ICS is of interest for its rarity and high degree of clinical relevance, especially in regard to surgical procedures in this region. This is a very rare case in which 5 CTs are present in one individual along with other arterial anomalies.

This case report adds to the literature on posterior thoracic wall arterial variations and gives a better understanding of the blood supply to the region. These potential variations should be considered by clinicians performing procedures in the thoracic cavity.
